# Predictive value of genomic screening: cross-sectional study of cystic fibrosis in 50,788 electronic health records

**DOI:** 10.1038/s41525-019-0095-6

**Published:** 2019-09-04

**Authors:** J. P. Sugunaraj, H. M. Brosius, M. F. Murray, K. Manickam, J. A. Stamm, D. J. Carey, U. L. Mirshahi

**Affiliations:** 1 Department of Pulmonary and Critical Care Medicine, Geisinger, Danville, PA USA; 2 Department of Genetics, Yale School of Medicine, New Haven, CT USA; 3 Division Genetic & Genomic Medicine, Nationwide Children’s Hospital, Columbus, OH USA; 4 Department of Molecular and Functional Genomics, Geisinger, Danville, PA USA

**Keywords:** Personalized medicine, Genetic techniques

## Abstract

Doubts have been raised about the value of DNA-based screening for low-prevalence monogenic conditions following reports of testing this approach using available electronic health record (EHR) as the sole phenotyping source. We hypothesized that a better model for EHR-focused examination of DNA-based screening is Cystic Fibrosis (CF) since the diagnosis is proactively sought within the healthcare system. We reviewed *CFTR* variants in 50,778 exomes. In 24 cases with bi-allelic pathogenic *CFTR* variants, there were 21 true-positives. We considered three cases “potential” false-positives due to limitations in available EHR phenotype data. This genomic screening exhibited a positive predictive value of 87.5%, negative predictive value of 99.9%, sensitivity of 95.5%, and a specificity of 99.9%. Despite EHR-based phenotyping limitations in three cases, the presence or absence of pathogenic *CFTR* variants has strong predictive value for CF diagnosis when EHR data is used as the sole phenotyping source. Accurate ascertainment of the predictive value of DNA-based screening requires condition-specific phenotyping beyond available EHR data.

## Introduction

Attempts to model genomic screening for long QT syndrome (LQTS) and arrhythmogenic right ventricular cardiomyopathy (ARVC), using electronic health record (EHR) as the lone source of phenotypic data has raised questions about the value of genomic screening more broadly in the accurate identification of genetic disease risk in low-prevalence cohorts.^1–4^

Cystic fibrosis (CF), like LQTS and ARVC, is relatively uncommon but it does not meet the NIH definition of a rare disease.^5^ The reported prevalence of these three conditions range from ~1 in 1250 to 1 in 3200.^6–8^

However, CF is distinct from LQTS and ARVC in that there has been over 20 years of extensive proactive CF diagnostic efforts to identify cases in adult, pediatric, and newborn care. These CF diagnostic efforts, incorporating both genetic and non-genetic diagnostic testing, have led to standardized diagnostic approaches to suspected cases of CF.^9^ As a result, the EHR of patients in major healthcare systems, contain a wealth of data from the diagnostic workups of patients for whom either clinical or laboratory findings have raised suspicion of the diagnosis.^10^

CF is an autosomal recessive disorder that affects one in 3200 in the United States and is caused by bi-allelic pathogenic variants (either homozygous or compound heterozygous pathogenic alleles) in the cystic fibrosis transmembrane conductance regulator (*CFTR*) gene.^11–13^ The most common pathogenic *CFTR* allele, F508del, accounts for ~70% of known CF-causing alleles.^14^

As non-DNA-based ascertainment is the first step toward diagnosis in this low-prevalence monogenic condition, CF provides a reasonable use case for examining the predictive value of DNA-based screening when available EHR data is the lone phenotypic source for case confirmation. Universal newborn screening for CF has been in place in the United states since 2010; all states use immunoreactive trypsinogen (IRT) as the initial screen followed by either repeat IRT (IRT/IRT) or DNA-based testing (IRT/DNA) as the follow-up screen.^15^ Separate from IRT-based newborn screening, a distinct second prompt for CF diagnosis is the presence of symptomatic disease.^9,16^ In either scenario, *CFTR* genetic testing has been employed as part of the diagnostic follow-up to another prompt for more than 20 years.

“Genome-first” describes a screening process that starts with detection of pathogenic DNA variants independent of any demographic or clinical information.^17^ Genome-first approaches to case identification of monogenic disease are increasingly feasible in large cohorts with genomic sequence data.^18–20^ Despite promising data regarding the use of this approach for two conditions with observed screen positive rates of 1 in 190 to 1 in 256 (i.e. *BRCA1/2* related cancer risk and Familial Hypercholesterolemia), the efforts cited above using genomic screening in this manner to identify less prevalent monogenic conditions have demonstrated suboptimal performance characteristics for case identification via this approach.^1,2^

A genome-first approach to case identification for autosomal recessive diseases in general, and to CF specifically, has not been previously examined in a large sequenced cohort. Given the existence of available diagnostic approaches and the anticipated robust diagnostic workup data of suspected cases in the EHR, we hypothesized that modeling a genotype-first screening approach to CF case identification using EHRs would perform better than previous efforts in LQTS and ARVC.

In this manuscript, we report the results of applying a genotype-first approach to CF case identification within the DiscovEHR dataset that links de-identified exome sequence data with participant’s de-identified EHR data.^21^ The data for this study came from 50,778 adult and pediatric participants in the DiscovEHR dataset recruited through the Geisinger MyCode Community Health Initiative (MyCode) in Pennsylvania. IRT-based newborn screening for CF began in Pennsylvania in 1995; therefore, any documented cases in individuals older than 23 years in Pennsylvania would be expected to have almost exclusively occurred through clinical presentations with signs or symptoms.

## Results

The DiscovEHR WES database (*n* = 50,778) was queried for CF-causing variants, defined as those variants identified in the CFTR2 database as CF-causal, and variants identified as pathogenic or likely pathogenic in the ClinVar database.

### Genome-first positives

**Fig. 1:**
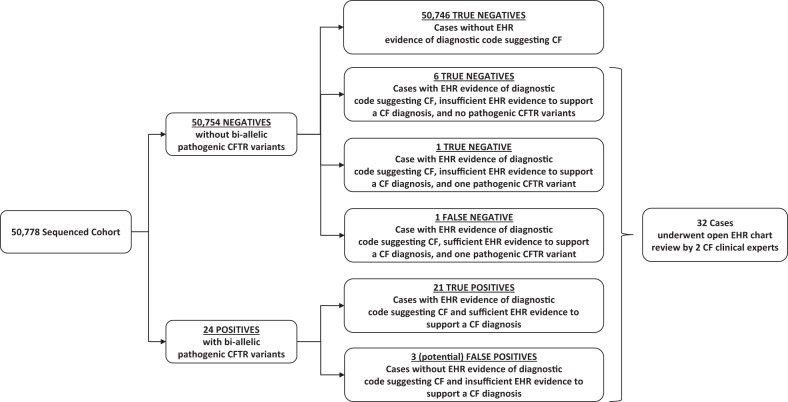
EHR-based Genomic Screening for CF. In a database of 50,778 participants, there were 50,754 negatives, namely cases without bi-allelic pathogenic *CFTR* variants, and 24 positives, namely cases with such variants. Amongst the negatives, eight had EHR data suggesting a CF diagnosis, six of those had no CFTR pathogenic variants, and two had a single pathogenic variant. Open chart review concluded that one of the heterozygotes was a false negative and one was a true negative. The remainder of the negatives, those without bi-allelic *CFTR* variants or EHR data consistent with CF were also considered true negatives. Open chart review was pursued for all 24 positive cases. The diagnosis of CF was confirmed in 21 cases. In the remaining three cases the diagnosis could not be confirmed due to insufficient available evidence

The exome sequence database query identified 24 cases with bi-allelic CF-causing *CFTR* variants (Fig. 1). Expert EHR review confirmed the diagnosis of CF in 21 individuals, who were then labeled true positives (TP); CF could not be confirmed definitively in three individuals (Table 1). This was due to insufficient evidence for, rather than evidence against, a CF diagnosis; these individuals were therefore labeled as “possible” false positives (FP).

**Table 1:** Details of cases undergoing open chart review

Patient #	CF Diagnosis	DNA screen positive? (Known pathogenic *CFTR* variants found by exome sequencing)	Diagnostic Category	Sex	Current age (years)	CF Diagnosis in EHR (age at diagnosis)^a^	Age of first encounter recorded in Geisinger EHR^a^	Sweat chloride test (mmol/L)	Clinical genetic testing	Clinical test concordant with WES	Bronchiectasis	Respiratory pseudomonas infection	Pancreatic supplement	Notations
1	Yes	Yes (F508del/F508del)	TP	M	6	Yes (0.3)	0	93	Yes	Yes	ND	ND	Yes	
2	Yes	Yes (F508del/F508del)	TP	F	19	Yes (3)	3	92	Yes	Yes	ND	ND	Yes	
3	Yes	Yes (F508del/F508del)	TP	F	20	Yes (4)	4	91	Yes	Yes	Yes	Yes	Yes	
4	Yes	Yes (F508del/F508del)	TP	F	21	Yes (4)	4	ND	Yes	Yes	ND	Yes	Yes	Transferred from another center; record of attempted sweat chloride testing reported as QNS
5	Yes	Yes (F508del/F508del)	TP	M	27	Yes (12)	12	112	Yes	Yes	Yes	Yes	Yes	
6	Yes	Yes (F508del/F508del)	TP	M	29	Yes (14)	14	90	Yes	Yes	ND	Yes	Yes	
7	Yes	Yes (F508del/F508del)	TP	F	30	Yes (15)	15	82	Yes	Yes	Yes	Yes	Yes	
8	Yes	Yes (F508del/F508del)	TP	M	34	Yes (29)	23	91	Yes	Yes	ND	ND	Yes	
9	Yes	Yes (F508del/F508del)	TP	F	37	Yes (18)	18	69	Yes	Yes	Yes	Yes	Yes	
10	Yes	Yes (F508del/F508del)	TP	F	38	Yes (30)	30	ND	Yes	Yes	Yes	Yes	Yes	Transferred from another center; results of sweat chloride testing not available
11	Yes	Yes (F508del/F508del)	TP	F	41	Yes (27)	27	97	Yes	Yes	Yes	Yes	Yes	
12	Yes	Yes (F508del/F508del)	TP	M	45	Yes (30)	29	ND	Yes	Yes	ND	ND	Yes	Transferred from another center; results of sweat chloride testing not available
13	Yes	Yes (F508del/F508del)	TP	M	54	Yes (38)	38	108	Yes	Yes	Yes	Yes	Yes	
14	Yes	Yes (F508del/F508del)	TP	F	55	Yes (47)	46	ND	No	NA	ND	Yes	Yes	Patient’s CF care at outside facility; results of clinical genetic testing and sweat chloride testing not available
15	Yes	Yes (F508del/Q39X)	TP	M	37	Yes (32)	27	ND	No	NA	ND	ND	ND	Patient’s CF care at outside facility; results of clinical genetic testing and sweat chloride testing not available
16	Yes	Yes (F508del/L88fs)	TP	M	6	Yes (1)	0.3	73	Yes	Yes	ND	Yes	Yes	
17	Yes	Yes (F508del/R117H T5/TG11)	TP	F	23	Yes (15)	7	ND	Yes	Yes	ND	ND	Yes	No sweat chloride testing done
18	Yes	Yes (c.489 + 1 G > T/c.3718–2477 C > T)	TP	F	37	Yes (19)	19	50	Yes	Yes	ND	Yes	Yes	
19	Yes	Yes (R334W/G542X)	TP	F	22	Yes (9)	6	103	Yes	Yes	ND	ND	Yes	
20	Yes	Yes (F508del/G551D)	TP	F	29	Yes (13)	13	90	Yes	Yes	ND	Yes	Yes	
21	Yes	Yes (F508del/K710X)	TP	M	45	Yes (34)	33	91	Yes	Yes	ND	ND	Yes	
22	Yes	No (F508del/ --)	FN	M	5	Yes (0.06)	0	66	Yes	Yes	ND	ND	Yes	Second variant = R1239S, not currently found in CFTR2 or ClinVar databases
23	No	No (F508del/ --)	TN	F	7	Yes (1)	0.3	<10, 14	Yes	Yes	ND	Yes	ND	Likely CRMS; second variant = S1235R, is “not CF-causing” or “benign” in databases
24	No	Yes (F508del/L206W^b^)	FP	M	62	No	46	ND	No	NA	ND	ND	ND	Unknown; insufficient data
25	No	Yes (F508del/L206W^b^)	FP	M	74	No	60	ND	No	NA	possible	ND	ND	Possible; insufficient data
26	No	Yes (F508del/Q1476X)	FP	F	68	No	53	ND	Yes/NA	No	Yes	Yes	ND	Possible; insufficient data; clinical genetic test in 2009 only reported F508del/−

CF diagnosis was assigned following open chart review by CF-expert clinicians independent of DNA-screening (see Materials and Methods). DNA-screening was positive or negative based on the presence or absence of bi-allelic pathogenic *CFTR* variants. DNA-screening positive was determined in 24 out of 26 reviewed cases; DNA-screening negative was determined in 2 out of 26 cases. The screen negative cases came to be reviewed because of the CF diagnostic codes in their EHR. Each case was placed in a diagnostic category, namely: true positive (TP), false positive (FP), true negative (TN), or false negative (FN). Open chart review was done on an additional six cases based on CF-associated ICD data; these charts were not found to have further evidence of CF and there were no pathogenic *CFTR* variants associated with these cases. *CRMS* CFTR-related metabolic syndrome, *ND* no data available, *NA* not applicable, *QNS* quantity not sufficient. ^a^Age in years when first recorded in EHR. ^b^Cases 24 and 25 found to have same rare pathogen variant (L206W, MAF 0.0003) together with F508del, but the two cases were confirmed through identity by descent to not be genetically related

### Genome-first negatives

The EHR-associated diagnostic codes for the 50,754 without bi-allelic pathogenic CFTR variants were queried. Two cases from this group had ICD codes consistent with a CF diagnosis and a mono-allelic *CFTR* pathogenic variant (Fig. 1). These cases underwent expert chart review which confirmed the diagnosis of CF in 1 who was then labeled a false negative (FN). Chart review concluded that the second case has *CFTR*-Related Metabolic Syndrome (CRMS) variant (Table 1) and was then labeled a true negative (TN). Six individuals had diagnosis codes for CF in EHR but did not harbor any pathogenic *CFTR* variants. Chart review indicated no evidence of CF and were labeled as true negatives. The remainder of the cases without bi-allelic CFTR variants and no ICD codes consistent with a CF diagnosis were also labeled as TN.

### Genetic variants in charts undergoing open review

Table 2 displays the details of the 14 *CFTR* variants relevant to these 26 cases. The F508del variant accounted for 73% of the 52 *CFTR* alleles. The cases discussed above that were ultimately identified as TN and FN were *CFTR* compound heterozygotes with F508del and S1235R (TN, with CRMS) and F508del and R1239S in the (FN). Importantly the S1235R variant has been judged non-pathogenic in both CFTR2 and ClinVar, and R1239S has not been evaluated for inclusion in either database. In addition to evidence of R1239S pathogenicity in case #22, there is additional bioinformatic support for the pathogenicity of this variant from our studies (see Table S2) and others.^22^

**Table 2:** *CFTR* variants found in 26 cases that underwent open chart review

	Variant cDNA name	Variant protein name		CFTR2 (8/31/18)	ClinVar Review Status^a^	ClinVar link (accessed 9/15/18)	# alleles in compound heterozygous	# alleles in homozygous	# alleles
1	c.115 C > T	p.Gln39Ter	Q39X	CF causing	Pathogenic *3	https://www.ncbi.nlm.nih.gov/clinvar/23273455/	1	0	1
2	c.263 T > G	p.Leu88Ter	L88X	CF causing	Pathogenic *3	https://www.ncbi.nlm.nih.gov/clinvar/variation/53534/	1	0	1
3	c.350 G > A	p.Arg117His	R117H	Varying clinical consequence	Pathogenic *4	https://www.ncbi.nlm.nih.gov/clinvar/RCV000007528/	1	0	1
4	c.489 + 1 G > T	NA	NA	CF causing	Pathogenic *4	https://www.ncbi.nlm.nih.gov/clinvar/RCV000043565/	1	0	1
5	c.617 T > G	p.Leu206Trp	L206W	CF causing	Pathogenic *3	https://www.ncbi.nlm.nih.gov/clinvar/RCV000007611/	2	0	2
6	c.1000 C > T	p.Arg334Trp	R334W	CF causing	Pathogenic *4	https://www.ncbi.nlm.nih.gov/clinvar/RCV000007559/	1	0	1
7	c.1521_1523delCTT	p.Phe508del	F508del	CF causing	Pathogenic *4	https://www.ncbi.nlm.nih.gov/clinvar/RCV000007523/	10	28	38
8	c.1624G > T	p.Gly542Ter	G542X	CF causing	Pathogenic *4	https://www.ncbi.nlm.nih.gov/clinvar/RCV000007535/	1	0	1
9	c.1652G > A	p.Gly551Asp	G551D	CF causing	Pathogenic *4	https://www.ncbi.nlm.nih.gov/clinvar/RCV000007540/	1	0	1
10	c.2128 A > T	p.Lys710Ter	K710X	CF causing	Pathogenic *3	https://www.ncbi.nlm.nih.gov/clinvar/RCV000007624/	1	0	1
11	c.3705 T > G	p.Ser1235Arg	S1235R	Non CF-causing	Benign	https://www.ncbi.nlm.nih.gov/clinvar/RCV000029527/	1	0	1
12	c.3717 G > C	p.Arg1239Ser	R1239S	NA	NA	NA	1	0	1
13	c.3718–2477 C > T	NA	NA	CF causing	Pathogenic *4	https://www.ncbi.nlm.nih.gov/clinvar/RCV000007586/	1	0	1
14	c.4426 C > T	p.Gln1476Ter	Q1476X	NA	Pathogenic *2	https://www.ncbi.nlm.nih.gov/clinvar/RCV000047135/	1	0	1
							*24*	*28*	*52*

Variants #11 (S1235R) and #12 (R1239S) were identified in cases 23 and 22, respectively (see Table 1), in each case forming *CFTR* compound heterozygotes with F508del; however, neither variant was found as pathogenic variants in either the CFTR2 or ClinVar databases. Variant #14 was missed on clinical testing obtained in 2009 for case 26 (see Table 1). Note that only 42% (5/12) of the pathogenic variants in this table were included in the 2004 ACMG panel #3, 6, 7, 8, 9. The genomic screening used in this manuscript (WES plus microarray) is more sensitive than many of the clinical genetic tests offered in the past. ^a^ClinVar review status: *2 = endorsed by two or more submitters providing assertion criteria provided the same interpretation, *3 = endorsed by expert panel, *4 = endorsed by practice guideline

The three possible FP cases are all F508del compound heterozygotes. The variant L206W found with F508del in two unrelated men over 60 years of age is considered pathogenic in both CFTR2 and ClinVar databases. The third possible FP case was a F508del/Q1476X compound heterozygote in a woman over 70 years of age; the Q1476X allele is described in ClinVar as pathogenic but is not present in CFTR2. Chart review documented that this patient underwent clinical *CFTR* testing in 2009 that revealed only the F508del heterozygous state. It was further noted that this patient’s clinical genetic testing at that time consisted of a limited evaluation for 23 known pathogenic variants; this evaluation was carried out 4 years prior to the documentation of Q1476X as a pathogenic variant in 2013 in ClinVar (see Table 2).

### Data concordance in clinical and research CFTR variant results

**Fig. 2:**
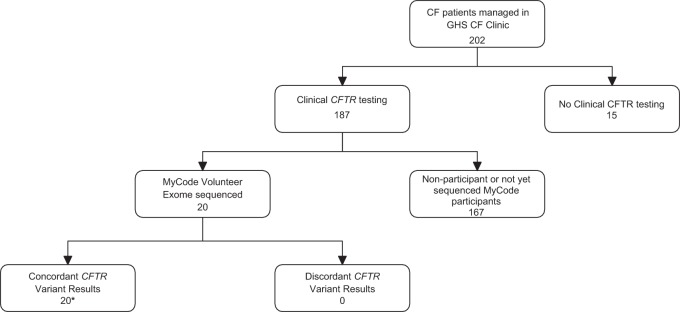
Data concordance in clinical and research *CFTR* variant results. Twenty of the 22 confirmed CF patients in the cohort also received their care in the Geisinger CF clinical program and had clinical CF genetic testing results available in their EHR. In the 20 cases with DNA data from both sources there was 100% pathogenic variant calling concordance

The concordance of clinical *CFTR* test results from individuals managed in Geisinger CF clinic and compared to exome sequencing results is displayed in Fig. 2. The 20 evaluable cases had 100% concordance.

### Predictive value of exome sequencing

Using the TP, FP, TN, and FN designations described above (displayed in Fig. 1 and Table 3), we calculated positive predictive value of 87.50% (69.23 to 95.61), negative predictive value of 99.99% (99.98–100.00), sensitivity of 95.45% (77.16–99.88), and a specificity of 99.99% (99.98–100.00).

**Table 3:** Screening predictive statistics

	Diagnostic category	
ES positive, *n* (%)		24 (0.05)
CF	TP	21
No CF	FP	3^a^
ES negative, *n* (%)		50,754 (99.95)
No CF	TN	50,753
CF	FN	1
Sensitivity, % (95% CI)		95.5 (77.2–99.9)
Specificity, % (95% CI)		99.99 (99.98–100.00)
PPV, % (95% CI)		87.5 (69.2–95.6)
NPV, % (95% CI)		99.99 (99.98–100.00)
Accuracy, % (95% CI)		99.99 (99.98–100.00)
Disease prevalence, % (95% CI)		0.04 (0.03– 0.07)

Individuals with or without CF are categorized as TP, FP, TN, or FN based on exome sequence variants previously classified as pathogenic. Screening predictive statistics are calculated as described in Methods. *ES* exome sequence, *CF* cystic fibrosis, *TP* true positive, *FP* false positive, *TN* true negative, *FN* false negative, *PPV* positive predictive value, *NPV* negative predictive value, *95% CI* 95% confidence interval. ^a^“potential” false positive – insufficient EHR data to rule in or rule out diagnosis

## Discussion

### CF cases in the MyCode Cohort

In the United States, the prevalence of CF in individuals of northern European background is 1:3200 (or 0.03%), with a carrier frequency of one in 28 individuals.^6^ The 22 confirmed CF cases in this MyCode cohort yields an empiric prevalence of 1:2300 (or 0.04%) individuals with CF, and we observed a carrier rate of one in 25 individuals (data not shown).

The positive predictive value (PPV) of screening tests are known to be influenced by the prevalence of the disease in the population that is being screened; more prevalent disease tends to correlate with higher PPV.^23^ It is therefore noteworthy that the population prevalence of CF, LQTS, and ARVC are estimated to be: CF 1:3200, LQTS 1:2500, and AVRC 1:1250.^6–8^ Although the authors did not calculate PPV in their manuscripts, a liberal estimate of PPV using the same definitions for EHR-based TP and FP would be 3.2% for LQTS and 0% for ARVC.^1,2^ A PPV of 87.5% was found for CF in our study.

This study carried out a genome-first DNA-based retrospective analysis of clinical data in a cohort with an average of 14 years of EHR data and an average age of 63 years. In this setting we demonstrated excellent predictive value for genetic screening-based identification of CF.

Newborn screening for CF is usually based on IRT, a non-DNA-based test. Kloosterboer et al. reported a sensitivity of 80.2% for the IRT/IRT screening algorithm.^24^ There have been numerous strategies proposed to improve the performance characteristics of the newborn CF screen.^25^ Vernooij-van Langen et al.^26^ carried out screening in 145,499 newborns in the Netherlands. The strategy that performed the best used two non-DNA screens (IRT and pancreatitis-associated-protein) followed by a two DNA screens (panel and sequencing) to get a sensitivity of 95.0%, a specificity of 100% and a PPV of 87.5%. The current study using DNA sequencing in a non-newborn cohort had a sensitivity, specificity, and PPV similar to the Vernooij-van Langen study.

Of the 26 potential cases reviewed with DNA data, 22 were classified as definite CF, three were not classified as CF due to insufficient data, and one was classified as CRMS. The individual with CRMS, was judge a TN. This case had absence of bi-allelic pathogenic *CFTR* variants and an EHR showing a negative sweat test result as part of a diagnostic CF workup. So although EHR diagnostic coding included a CF diagnosis, that has been revised to CRMS. It is noteworthy that the DiscovEHR exome sequence data, array genotype data, and clinical genetic tests results, when available, were completely concordant.

The exome sequence data identified three possible previously undiagnosed CF cases that were conservatively judged FP (see evidence support pathogenicity for these variants with milder disease phenotypes in Bioinformatic Narrative in the supplements). In one of these individuals there had been clinical suspicion of CF and a record of completed clinical genetic testing for CF at an outside institution. The other two (cases 24 and 25) had no evidence of clinical testing for CF in their EHR, but exome sequencing identified bi-allelic pathogenic variants (F508del and L206W), which have been classified as a CF-causing combination in both the CFTR2 and ClinVar databases.

### Improving variant databases

This work relied on the existence of robust variant interpretation databases, and the importance of maintaining and expanding these datasets was highlighted by two of the cases. The clinical testing of case 26 with the heterozygous combination of F508del and Q1476X in 2009 failed to identify a second pathogenic allele; her second pathogenic allele identified in this study was first entered into public data in 2013.

One previously not observed variant that we encountered was R1239S; this variant was found in combination with F508del in case 22 (designated FN). Designation of R1239S as a newly recognized pathogenic variant is supported by our patient’s CF diagnosis as well as the bioinformatics analysis in Table S2, and its addition to ClinVar through this work may help future variant interpretation.

CF is a disease that is most prevalent in people of European ancestry, and the MyCode cohort has 98% European-Americans (see Table S1). It will be important to seek replication of our findings for *CFTR*-CF in more diverse cohorts where there is a greater potential for additional pathogenic variants not currently in the databases.

### Modeling genome-first population screening using EHRs

Growing enthusiasm for using DNA-based screening is tempered by legitimate concerns that this could lead to over-diagnosis and over-estimates of risk.^27^ As discussed above, some early attempts to use DNA-based screening of EHR data in cardiac genetic conditions appear to lend support to these concerns. However, the landmark publications on LQTS and AVRC highlight significant limitations linked to those conditions, including: (1) expert disputes over what to interpret as pathogenic genetic variants, (2) a lack of targeted condition-specific diagnostic evaluations in the EHR data associated with cases presenting with partial phenotypes (i.e. minor diagnostic criteria), and (3) the likelihood that we currently have an incomplete understanding of condition-specific genotype-phenotype correlations (i.e. questions of critical environmental factors for ARVC).^1,2^

With regard to these limitations in the setting of these two cardiogenetic conditions, the first is being overcome through efforts such as ClinVar and ClinGen,^28^ the second can be overcome in clinical settings where patients are called back for more extensive phenotyping,^29^ and the third will require more research into basic genotype-phenotype correlation.^2^

In the case of CF, examined in this work, the limitations observed in the LQTS and ARVC examples are largely overcome through the use of: expert consensus on pathogenic *CFTR* variant calls, a clinical data source (i.e. Geisinger’s EHR) where comprehensive diagnostic phenotyping and clinical genetic testing of suspected cases is common, and a gene-disease example with clearly established diagnostic criteria that are routinely interrogated by expert clinicians in suspected cases. In this setting there is little evidence of over-estimate of CF risk given the finding that 87.5% (21 out of 24) of those who screened positive had confirmed CF, and there is little expectation of over-diagnosis since the remaining cases who lack sufficient diagnostic evidence could potentially undergo a clinical evaluation and secondary screening with sweat chloride testing to reach a clear conclusion.

Prior to this CF example, given the data on LQTS and AVRC, it would have been tempting to conclude that poor PPVs were inescapable when pursuing population-based DNA-screening for monogenic disease risk in unselected patient groups. However, since this work demonstrates that a desirable PPV can be attained for CF, further research focused on a deeper examination of the factors surrounding these three conditions that result in dramatic differences in PPV should be pursued.

CF is a particularly well-studied monogenic condition. CF is a recessively inherited disorder with nearly 100% penetrance in homozygous or compound heterozygous individuals. Proactive efforts aimed at case identification and diagnosis have occurred within healthcare for more than two decades. A large number of pathogenic variations in the *CFTR* gene are known, the molecular consequences of those changes are understood, and expertly curated publically available databases containing these variants exist. The sweat chloride test has been in use for 60 years.^9,30,31^ This routinely employed test provides non-DNA-based laboratory evidence of *CFTR* dysfunction and can be employed to confirm CF diagnoses. CF is clinically distinct without commonly encountered phenocopies.^32^ The range of clinical manifestations of CF including the mild end of the spectrum is well studied.

Together these features have allowed us to test genome-first screening through a cross-sectional study design using the Geisinger EHR, which has been in place for over 20 years, as the sole clinical data source.

With this design and in this setting, we were able to measure both the PPV and the NPV, as well as the sensitivity and specificity of using DNA variant detection as a primary screening technique. Our strict definition of FP led to 3 FP cases amongst 24 positives, even though 2 of those 3 are probable CF and would likely be converted to TP on follow-up evaluation. Therefore, the measured PPV of 87.5% for genome-first case detection of CF in this cohort is perhaps mildly suppressed by our retrospective EHR-based study design and demonstrates the need for condition-specific phenotyping beyond the available EHR data for incidental or secondary genetic findings.

CF likely constitutes a gold-standard for the performance of genome-first case identification for monogenetic conditions in population cohorts where the individuals tested have a low prior probability. It is likely that the prospective use of genome-first screening will vary in its diagnostic accuracy and predictive value depending on the cohort and the evidence-base surrounding the chosen gene-condition pair. Importantly, the application of genome-first screening will need to accommodate for additional variables such as age-related penetrance (e.g. for cancer or heart disease) in those gene-condition pairs where it is a major factor and there are no secondary clinical tests of gene function such as the sweat chloride test.

It seems likely that an evidence-based genome-first gene-condition pair list will be a more restricted list of gene-condition pairs compared to the list for secondary findings within data generated for unrelated diagnoses.^33,34^ Minimum gene-condition test performance standards may be needed in order to support the employment of genome-first screening outside of the research setting. The development of expert consensus criteria could help establish when a gene-condition pair has the sufficient evidence-base to support population screening.

Since there is an existing population screen for CF that is effective and does not use DNA as the primary screening tool, CF may be amongst the conditions least likely to see significant changes in diagnoses if a DNA-based population screening were pursued. However, using CF as an exemplar in this finding, we demonstrate that an accurate ascertainment of the predictive value of DNA-based screening requires condition-specific phenotyping beyond available EHR data.

## Methods

### Cohort description

The study cohort consisted of individuals who consented to participate in MyCode, an Institutional Review Board (IRB)-approved program to create a biorepository of blood, serum, and DNA samples for broad research use, including genomic analysis. This study was reviewed by the Geisinger IRB and determined to not be human subjects research as defined in 45CFR46.102(f) in written consent (Study #2017–054). Data obtained from analysis of MyCode samples is linked to Geisinger EHR; study participants have a median of 14 years of EHR data, providing a longitudinal database of clinical diagnosis, procedures, medications, and laboratory results. As part of the DiscovEHR collaboration between Geisinger and the Regeneron Genetics Center, DNA samples undergo microarray genotyping and whole exome sequencing (WES). The data analyzed for this study consisted of exome variants from the first 50,778 participants, including children. The cohort characteristics have been described previously^18^ and are summarized in Table S1. This study was approved by the Geisinger Institutional Review Board (IRB).

### Genotype analysis

WES for the DiscovEHR collaboration has been described previously.^21,35^ WES variants were confirmed using genotyping data when the markers were available. The same DNA samples were genotyped using the Infinium OmniExpressExome microarray Beadchip (Illumina, San Diego, CA). Following project level quality controls (genotype and sample call rates >98%, Hardy-Weinberg equilibrium *p* > 1e-06, and allelic counts greater than or equal to 2), 59,273 samples and 889,966 variants remained for downstream analysis. Of the *CFTR* variants identified in the patients in the present study, a subset (Q39X, R117H, L206W, G542X, G551D, S1235R, c.489 + 1 G>T, and c.3718–2477 C>T) was available as markers on the BeadChip; all samples with markers on the BeadChip confirmed WES calls, providing a confirmation of the patient genotype status.

### Variant annotation and pathogenicity assessment

Sequence variants were annotated to coding DNA and functional proteins using the NCBI RefSeq Gene definitions, selecting for the transcript with the longest coding sequence among the transcripts with a Locus Reference Genome (LRG) annotation, and excluding transcripts without annotated start and stop codons (SNP & Variation Suite, Golden Helix, Bozeman, MT).^36,37^ All variants were transcribed to RefSeq NM_000492.3 and translated to NP_000483.3.

The Clinical and Functional Translation of *CFTR* database (CFTR2, as of 31 August 2018) and the ClinVar database (as of September 15, 2018) were used as the source of validated pathogenic and non-pathogenic *CFTR* variants.^38,39^

### EHR diagnostic code review

CF ICD10 codes (E84.0, CF with pulmonary manifestations; E84.11, meconium ileus in CF; E84.19, CF with other intestinal manifestations; E84.8, CF with other manifestations; E84.9, CF, unspecified) identified all EHRs among the 50,778 participants who had a relevant CF diagnosis. This list was cross referenced with the list of cases with bi-allelic pathogenic *CFTR* variants; cases not previously identified through the variant process were sent for open chart review by 2 CF experts (described below) to adjudicate their CF diagnosis.

### Clinical open chart review

Twenty-six cases with either pathogenic bi-allelic *CFTR* variants identified by DNA analysis (WES plus array), or a CF diagnosis in the EHR without pathogenic bi-allelic *CFTR* variants underwent open chart review by experienced CF clinicians (CF physician and CF program nurse coordinator). All relevant clinical data were extracted from the EHR, including clinical history as available in progress notes, diagnoses, biochemical parameters, commercial genetic testing, prescription history, spirometry results, and radiographic studies. CF diagnoses was confirmed when an individual had both a clinical presentation of the disease and evidence of *CFTR* dysfunction or abundant clinical evidence of CF.

CF was also considered present in the following: [1] *CFTR* dysfunction, defined as sweat chloride >60 mmol/L, and clinical *CFTR* genetic analysis showing a combination of two CF-causing *CFTR* pathogenic variants found in CFTR2 or ClinVar, as per current guidelines from the CF Foundation, [2] if absent sweat chloride testing then CF diagnosis required genetics that were consistent with CF causing pathogenic variants and consistent clinical features, such as longitudinal care by CF physicians, bronchiectasis, and exocrine pancreatic insufficiency. No diagnosis was assumed in those patients lacking sufficient clinical information, irrespective of genetic results.

### Concordance of CFTR variant identification in clinic testing and research exome testing

The list of patients currently managed in the Geisinger CF clinic and their clinical genetic test results were extracted from EHR. The subset who were sequenced and genotyped as volunteers in MyCode were identified. Correlation of variant findings in clinical testing and research sequencing was determined. Clinical genetic testing data was not available on cases of confirmed CF where disease testing and management was carried out at a non-Geisinger facility.

### Statistical analysis and graphs

Standard statistical analyses and all data were plotted using GraphPad Prism (La Jolla, CA).

## Supplementary information

Supplemental Material

## Data Availability

All data generated or analyzed during this study are included in this published article (and its supplementary information files).

## Supplementary information

**Supplementary information** accompanies the paper on the *npj Genomic Medicine* website (10.1038/s41525-019-0095-6).
