# Supplementary material for: Predictive value of genomic screening: cross-sectional study of cystic fibrosis in 50,788 electronic health records
Source: NPJ Genom Med. 2019 Sep 4;4:21. doi: 10.1038/s41525-019-0095-6 (PMC6726623; doi:10.1038/s41525-019-0095-6)
Supplement: Supplementary file 1 — Supplemental Material [file 41525_2019_95_MOESM1_ESM.pdf]

Online Supplements

Predictive Value of Genomic Screening: Cross-sectional Study of Cystic Fibrosis in 50,788 Electronic Health Records

Sugunaraj JP[1], Brosius HM[1], Murray MF[2], Manickam K[3], on behalf of the DiscovEHR Collaboration, Stamm JA[1*], Carey DJ[4*], and Mirshahi UL[4*]

Online Supplemental Tables and Figures

Supplemental Table 1. Demographic characteristics of CF-diagnosed case and control individuals in the study.

Supplemental Table 2. Splice-man prediction of exonic variant effect on splicing variants.

Supplemental Discussion. Clinical and Bioinformatic Narrative

Supplemental References

Supplemental Table 1. Demographic characteristics of CF-diagnosed case and control individuals in the study. Cases are confirmed per clinician chart-review, and controls are individuals without CF diagnosis or individual carriers of 1 or no *CFTR* pathogenic variants.

	CF-Diagnosed	No-CF Controls	p Values*
Number of individuals	22	50,756	
% Caucasian	100	98	
% Smokers	5	40	
Sex (% females)	55	59	0.1
Median age (range)	29 (4-55)	63 (2-89)	<0.001
Median height, inches (range)	64 (43-72)	66 (28-101)	0.2
Median lifetime maximum BMI (range)	23 (18-36)	34 (14-68)	<0.0001

* Two-tailed p values, Student's t-test followed by Mann-Whitney non-parametric post-hoc.

Abbreviations: CF, cystic fibrosis; BMI, body mass index.

Supplemental Table 2. Spliceman prediction of exonic variant effect on splicing variants. Intra-allelic distance (L1) and L1 ranking was generated for the hexamer mRNAs around the point mutations of c.3717G ("Wildtype" in capital font) to different nucleotides ("Mutation" in capital font) as in Lim *et al*.). Higher ranking indicates the higher likelihood of the variant in altering the splicing activity of the nearby canonical splice site. The variant R1239S (c.3717G>T) is observed in Patient #22 with CF (arrow). Values for two variants that were shown by molecular biology methods to cause aberrant splicing, *ABCB1* Thr3587Gly and *TYR* Thr1315Gly, are shown for comparison[1].

Gene	Protein	CFTR2 Classification	Point mutation	Wildtype	Mutation	L1 Distance	Ranking (L1)
CFTR	R1239R	CF-causing	cagag(g/a)gtggg	Ggtggg	Agtggg	35593	70%
CFTR	R1239S	?	cagag(g/c)gtggg	Ggtggg	Cgtggg	37639	81%
→ CFTR	R1239S	?	cagag(g/t)gtggg	gGgtgg	gTgtgg	34907	67%
ABCB1	T3587G	NA	tcata(t/g)tttgc	taTttt	taGttt	40302	94%
TYR	T1315G	NA	tcctc(t/g)tggca	tcctcT	tcctcG	37588	80%

Abbreviations: mRNAs, message RNAs; CF, cystic fibrosis; NA, not applicable; CFTR2, the Clinical and Functional TRanslation of CFTR database

Supplemental Discussion

Clinical Narrative

CF Diagnosed Cases (#1-22 in Table 1): Twenty-two patients who underwent open chart review and had either one or two pathogenic *CFTR* variant in the variant databases had a confirmed CF diagnosis.

Sweat chloride data was available and was consistent with CF in 12 patients (55%). Radiographic bronchiectasis was identified in seven patients (32%), and 13 (59%) had respiratory cultures with *Pseudomonas aeruginosa* colonization, clinical features of CF. Twenty individuals (91%) had a clinical genetic screening test performed, with results available in the EHR. In every case, the clinical genetic test results were consistent with the exome sequence analysis. Only two individuals (patients #14 and #15) had neither sweat chloride test nor clinical genetic test data available in the Geisinger EHR. On chart review both individuals were determined to have obtained most of their medical care at non-Geisinger facilities; both had bi-allelic pathogenic variants and were prescribed CF-specific medications by outside CF care centers, consistent with a diagnosis of CF.

To summarize the results for patients #1-22, all these individuals were deemed after expert chart review to have a correct diagnosis of CF. Through WES, F508del is identified in 20 of 22 (91%) of CF cases in our cohort (64% homozygotes and 27% compound heterozygotes). The *CFTR* variant combinations found in 21 of these 22 confirmed CF cases were previously reported in the CFTR2 database to be CF-causal. We identified a compound heterozygous carrier with genotype F508del/R1239S who was determined to

have CF based on abnormal sweat chloride test results and pancreatic insufficiency, suggesting R1239S is a novel CF-causing variant.

CF Not Diagnosed Cases (#23-26 in Table 1):

The diagnosis of CF could not be confirmed four individuals with either one or two pathogenic variant who underwent open chart review. Three were found to have two potential CF-causing *CFTR* variants by exome sequencing but lacked a documented history of CF in the EHR. Two of these individuals (patients #24 and #25) had *CFTR* genotype F508del/L206W, which has been reported to be a CF-causal combination in the CFTR2 database. One (#25) of these had a history of chronic lung disease and possible bronchiectasis suggestive of CF but is deceased. The other individual (#24) had insufficient information in his health record to make a determination. Patient #26 had the genotype combination F508del/Q1476X, which has not been previously reported in CFTR2. She has undergone an inconclusive clinical workup for CF including clinical *CFTR* testing that confirmed a single F508del allele; however, the *CFTR* screening panel in 2009 did not include Q1476X.

One individual (patient #23, a seven-year-old female) had an existing CF diagnosis and one pathogenic *CFTR* variant and one benign variant confirmed by exome sequencing and clinical genetic testing. This individual has a *P. aeruginosa*-positive respiratory culture and no indication of bronchiectasis. Results for sweat chloride tests were not consistent with CF. The genotype (F508del/S1235R) of this patient was previously reported to be associated with *CFTR*-Related Metabolic Syndrome (CRMS)[1].

To summarize results for patients #23-26, three individuals were identified with previously identified bi-allelic CF-causing variants; on chart review, one was classified as possible CF; there was insufficient information in the EHR to classify the other two patients. One individual was deemed to have symptoms consistent with CRMS, not CF.

Bioinformatic Narrative

The exome sequence data identified three possible previously undiagnosed CF cases, namely three individuals with bi-allelic pathogenic *CFTR* variants. None of these individuals had a record of complete clinical genetic testing for CF. Two (patient #24 and #25) had the F508del/L206W genotypes from exome sequencing, which has been classified as a CF-causing combination in the CFTR2 database. The other had a F508del and Q1476X (NM_000492.3:c.4426C>T; patient #26); Q1476X is in ClinVar but not in the CFTR2 database. Q1476 is the 5th to last amino acid in the c-terminus of *CFTR*. The c-terminal domain of *CFTR* is highly conserved in mammals and fish [2]; the prevalence of Q1476X is 1.029e-4 in the DiscovEHR study and 2.512e-5 in gnomAD [3]. The c-terminus of *CFTR* is critical for post-translational processing and polarization of *CFTR*, and biochemical and molecular studies showed that truncation of these domains lead to aberrant *CFTR* expression and function [4-6]. Four individuals with compound heterozygosity of Q1476X have been previously reported, incidentally all with F508del as the other variant. Lucarelli *et al*. described a patient from the Italian CF Reference Center who presented normal sweat chloride concentrations and no CF-related clinical features and classified the variant as CRMS [7]. Others reported F508del/Q1476X patients with elevated sweat chloride (>60 mM) and normal spirometry with no obvious CF-related

pancreatic or lung diseases [8, 9]. Likewise, compound heterozygotes of a CF-causal variant and a truncation variant (including large deletions) at the c-terminus of *CFTR* also presented with milder clinical features of CF, specifically, elevated sweat chloride but no pulmonary complications observed in CF [9, 10]. Retrospective chart-review of the individual harboring F508del/Q1476X variants discovered through exome sequencing in our study provided evidence of *P. aeruginosa*-positive respiratory cultures, normal spirometry, and radiographic report of bronchiectasis. However, further phenotyping including sweat chloride tests is required to definitively confirm the CF diagnosis as this patient's records indicate she was receiving specialty care outside our hospital system.

The other two (cases 24 and 25) had no evidence of clinical testing for CF in their EHR, but exome sequencing identified bi-allelic pathogenic variants (F508del and L206W), which have been classified as a CF-causing combination in both the CFTR2 and ClinVar databases. That these patients are 62 and 74 years old and lack a CF diagnosis suggests the L206W variant is not fully penetrant or is associated with heterogeneous phenotypic expression and a mild form of the disease in some individuals. Consistent with this, Desgeorges and colleagues reported 4 French patients with F508del/L206W variants who presented with pancreatic sufficiency and residual channel function.[11] Similarly, of the 235 CF patients in CFTR2 with F508del/L206W genotype, 90% are pancreatic sufficient, 78% negative for *P. aeruginosa*, and 92% had normal lung functions.[12] Sweat chloride tests showed slightly elevated levels (mean ~70 mEq/L vs. 100 mEq/L for all 59,324 CF patients in the database).

Our findings also provide evidence for another novel CF-causal variant, R1239S (NM_000492.3:c.3717G>T). R1239S is the last amino acid of exon 22, in the Nucleotide

Binding Domain 2 (NBD2) of *CFTR.* NBD2 is essential for *CFTR* channel gating, dimerization with NBD1, and channel open time [13]. NBD2 mutations are thought to prolong *CFTR* channel opening, although cross-talks between NBDs suggest that mutations in these domains affect the overall ATPase activity of *CFTR* [14]. In addition to its role in NBD in channel gating, the c.3717 position is also adjacent to the splicing junction. Using CryptSplice, Lee and colleagues showed that substitution of the guanine (G) nucleotide at this position to an adenine (A) or a cystosine (C), regardless of amino acid changes, reduced the splicing activity of the canonical splice donor by almost 30% [15]. Using Spliceman [16], we showed that the c.3717G>T variant modestly alters the splicing activity of the canonical splice donor site (Supplemental Table 2). This suggests that this locus is very sensitive to sequence changes that can alter recognition of the canonical splice site, possibly altering the splicing of the pre-mRNA and disrupting proper *CFTR* processing. Indeed, synonymous and exonic variants of *CFTR* have been shown to alter *CFTR* splicing activity [16, 17]. Lastly, the R1239R (NM_000492.3:c.3717G>A, synonymous) variant at the same codon position in combination with F508del has been reported to be CF-causal in two patients in the CFTR2 database. These findings and our clinical diagnosis strongly suggest that R1239S (c.3717G>T) is a novel CF-causal variant. The molecular mechanism through which this variant alters *CFTR* function remains to be determined but is an important consideration in pharmacotherapy.

Narrative Summary

In the United States, the prevalence of CF in Non-Hispanic Whites ranges from 1:3000 to 1:2500, with a carrier frequency of one in 29 individuals [18, 19]. A conservative estimate of

the 22 confirmed CF cases in our cohort yield a prevalence of 1:2300 (or 4%) individuals with CF and a carrier rate of one in 25 individuals (data not shown). The likelihood of a missed CF diagnosis is reduced substantially by universal newborn screening, which has been implemented in nearly all states in the US since 2010 [20]. Individuals born before the introduction of universal screening or who for various reasons did not undergo screening at birth could have been missed. In these individuals, variability of *CFTR* gene dysfunction, age of disease onset, and severity of organ dysfunction could lead to delayed or missed diagnosis. Additionally, increasing availability of exome and genome sequencing will likely uncover new *CFTR* variants and expand the spectrum of clinical CF. Indeed, our study demonstrates that exome sequence data from an unselected clinical population can identify individuals with pathogenic *CFTR* variants who appear to lack a previous clinical diagnosis. It is interesting to note that the three individuals with no previous CF diagnosis but suggestive genotypes, and in two of the cases supporting clinical data, are age 62 or older. Further evaluation of these patients to either confirm or rule out a CF diagnosis was not possible given our currently approved IRB protocol, but may have allowed for confirmation of one or more of these cases.

Supplemental References

1. Monaghan, K. G. *et al*. Frequency and clinical significance of the S1235R mutation in the cystic fibrosis transmembrane conductance regulator gene: results from a collaborative study. *Am. J. Med. Genet.* **95**, 361-365 (2000).

2. Chen, J. M. *et al*. A combined analysis of the cystic fibrosis transmembrane conductance regulator: implications for structure and disease models. *Mol. Biol. Evol.* **18**, 1771-1788 (2001).

3. Lek, M. *et al*. Analysis of protein-coding genetic variation in 60,706 humans. *Nature* **536**, 285-291 (2016).

4. Milewski, M. I., Lopez, A., Jurkowska, M., Larusch, J. & Cutting, G. R. PDZ-binding motifs are unable to ensure correct polarized protein distribution in the absence of additional localization signals. *FEBS Lett.* **579**, 483-487 (2005).

5. Sharma, N. *et al*. A sequence upstream of canonical PDZ-binding motif within CFTR COOH-terminus enhances NHERF1 interaction. *Am. J. Physiol. Lung Cell. Mol. Physiol.* **311**, L1170-L1182 (2016).

6. Moyer, B. D. *et al*. A PDZ-interacting domain in CFTR is an apical membrane polarization signal. *J. Clin. Invest.* **104**, 1353-1361 (1999).

7. Lucarelli, M. *et al*. A Genotypic-Oriented View of CFTR Genetics Highlights Specific Mutational Patterns Underlying Clinical Macrocategories of Cystic Fibrosis. *Mol. Med.* **21**, 257-275 (2015).

8. Claustres, M. *et al*. CFTR-France, a national relational patient database for sharing genetic and phenotypic data associated with rare CFTR variants. *Hum. Mutat.* **38**, 1297-1315 (2017).

9. Bienvenu, T. *et al*. Mutations located in exon 24 of the CFTR gene are associated with a mild cystic fibrosis phenotype. *Clin. Genet.* **64**, 266-268 (2003).

10. Mickle, J. E. *et al*. A mutation in the cystic fibrosis transmembrane conductance regulator gene associated with elevated sweat chloride concentrations in the absence of cystic fibrosis. *Hum. Mol. Genet.* **7**, 729-735 (1998).

11. Desgeorges, M., Rodier, M., Piot, M., Demaille, J. & Claustres, M. Four adult patients with the missense mutation L206W and a mild cystic fibrosis phenotype. *Hum. Genet.* **96**, 717-720 (1995).

12. http://cftr2.org.

13. Zhang, Z. & Chen, J. Atomic Structure of the Cystic Fibrosis Transmembrane Conductance Regulator. *Cell* **167**, 1586-1597.e9 (2016).

14. Zhang, Z., Liu, F. & Chen, J. Conformational Changes of CFTR upon Phosphorylation and ATP Binding. *Cell* **170**, 483-491.e8 (2017).

15. Lee, M. *et al*. Systematic Computational Identification of Variants That Activate Exonic and Intronic Cryptic Splice Sites. *Am. J. Hum. Genet.* **100**, 751-765 (2017).

16. Lim, K. H. & Fairbrother, W. G. Spliceman--a computational web server that predicts sequence variations in pre-mRNA splicing. *Bioinformatics* **28**, 1031-1032 (2012).

17. Ramalho, A. S. *et al*. Comparative ex vivo, in vitro and in silico analyses of a CFTR splicing mutation: Importance of functional studies to establish disease liability of mutations. *J. Cyst Fibros* **15**, 21-33 (2016).

18. Nakano, S. J. & Tluczek, A. Genomic breakthroughs in the diagnosis and treatment of cystic fibrosis. *Am. J. Nurs.* **114**, 36-43; quiz 44-5 (2014).

19. Abou Tayoun, A. N. *et al*. A comprehensive assay for CFTR mutational analysis using next-generation sequencing. *Clin. Chem.* **59**, 1481-1488 (2013).

20. Farrell, P. M., White, T. B., Derichs, N., Castellani, C. & Rosenstein, B. J. Cystic Fibrosis Diagnostic Challenges over 4 Decades: Historical Perspectives and Lessons Learned. *J. Pediatr.* **181S**, S16-S26 (2017).
